# Development and validation of the novel subclassification of pN3 for patients with esophageal cancer

**DOI:** 10.3389/fonc.2023.1113711

**Published:** 2023-05-02

**Authors:** Keru Ma, Hao Wang, Chengyuan Fang, Xiangyu Jiang, Jianqun Ma

**Affiliations:** ^1^Department of Thoracic Surgery, Esophagus and Mediastinum, Harbin Medical University Cancer Hospital, Harbin, China; ^2^Department of Gastroenterological Surgery, Harbin Medical University Cancer Hospital, Harbin, China

**Keywords:** esophageal cancer, pN3 stage, SEER, prognosis, lymph node metastases

## Abstract

**Background:**

Patients with stage pN3 esophageal cancer (EC) have a large number of metastatic lymph nodes (mLNs) and have poor prognosis. This study was to elucidate whether subclassification of pN3 according to the number of mLNs could improve the discrimination ability of EC patients.

**Methods:**

This study retrospectively analyzed patients with pN3 EC from the Surveillance, Epidemiology, and End Results (SEER) database as a training cohort and SEER validation cohort. Patients with pN3 esophageal cancer from the Affiliated Cancer Hospital of Harbin Medical University were used as the validation cohort. The optimal cutoff value of mLNs was identified using the X-tile software, and group pN3 into pN3-I and pN3-II based on mLNs. Kaplan-Meier method and log-rank test were used to analyze the disease-specific survival (DSS). The Cox proportional hazards regression analysis was used to identify the independent prognostic factors.

**Results:**

For the training cohort, patients with 7 to 9 mLNs were categorized as pN3-I, while those with more than 9 mLNs were categorized as pN3-II. There were 183 (53.8%) pN3-I and 157 (46.2%) pN3-II. The 5-year DSS rates of pN3-I and pN3-II in the training cohort were 11.7% and 5.2% (*P*=0.033), and the pN3 subclassification was an independent risk factor associated with patient prognosis. More RLNs may not improve patient prognosis, but the use of mLNs/RLNs is effective in predicting patient prognosis. Furthermore, the pN3 subclassification was well validated in the validation cohort.

**Conclusion:**

Subclassification of pN3 can better distinguish survival differences in EC patients.

## Introduction

Esophageal cancer (EC) has the seventh highest incidence and is the sixth leading cause of cancer death, causing approximately 509,000 deaths every year (1). Lymph nodes (LNs) metastasis of tumor cells is an important predictor of survival and recurrence in EC patients. It has been reported that LNs metastasis occurs in approximately 22%-43% of EC patients (2–4), and the 5-year survival rate of EC patients is less than 35% when it occurs (5). LNs metastasis is also an independent risk factor related to the prognosis of EC patients (6). Therefore, accurate identification of LN metastasis (mLNs) is crucial in predicting the prognosis of EC patients and developing effective treatment strategies.

The American Joint Committee on Cancer (AJCC) and the International Union for International Cancer Control (UICC) classified pN staging into pN0 stage and pN1 stage according to the regional mLN status in the sixth edition of the TNM staging system (7). To more accurately predict patients’ prognosis, according to the seventh edition of the AJCC-UICC classification system, pN is categorized into pN0 (0 mLNs), pN1 (1~2 mLNs), pN2 (3~6 mLNs), and pN3 (≥7 mLNs), which is a more specific classification based on the number of mLNs (8), and the predictive performance of the seventh edition classification was better than the sixth (9). In addition, the classification of the 7th edition of pN staging is still retained in the 8th edition of AJCC staging. This suggests that the number of mLNs is important for patient prognosis, and that detailed classification of pN stages according to mLNs can better individualize the risk stratification of patients.

pN3 disease characterized by extensive lymph node metastasis portends extremely poor prognosis (10). For mLNs with a wide range of pN3 stage, regardless of any pT stage, the final pTNM stage of EC patients of any stage pN3 stage was included in stage IV along with patients with distant metastases, which indirectly suggested that pN3 stage produces similar prognostic effects in EC patients with different disease progression (11). However, few studies on pN3 have been conducted because of the limited number of pN3 patients worldwide. In Asia, pN3 patients account for only 11.2%-19.4% of all EC patients (12, 13). In Western countries, pN3 patients account for only 4.6%-13.0% of the total patients (5, 9, 14). Given the limited number of relevant studies, it is unclear whether such patients have different disease progression. Therefore, further studies are needed to develop a novel subclassification for pN3 patients to refine the risk stratification to determine differences in survival among EC patients.

This study used the Surveillance, Epidemiology, and End Results (SEER) database to evaluate the prognosis of patients with pN3 stage and explore differences in survival among such patients. We attempt to clarify whether the subclassification of pN3 stage can be a better risk stratification for patients. In addition, we validated the results using patients with stage pN3 EC from the Cancer Hospital Affiliated to Harbin Medical University.

## Materials and methods

### Patients

**Figure 1:**
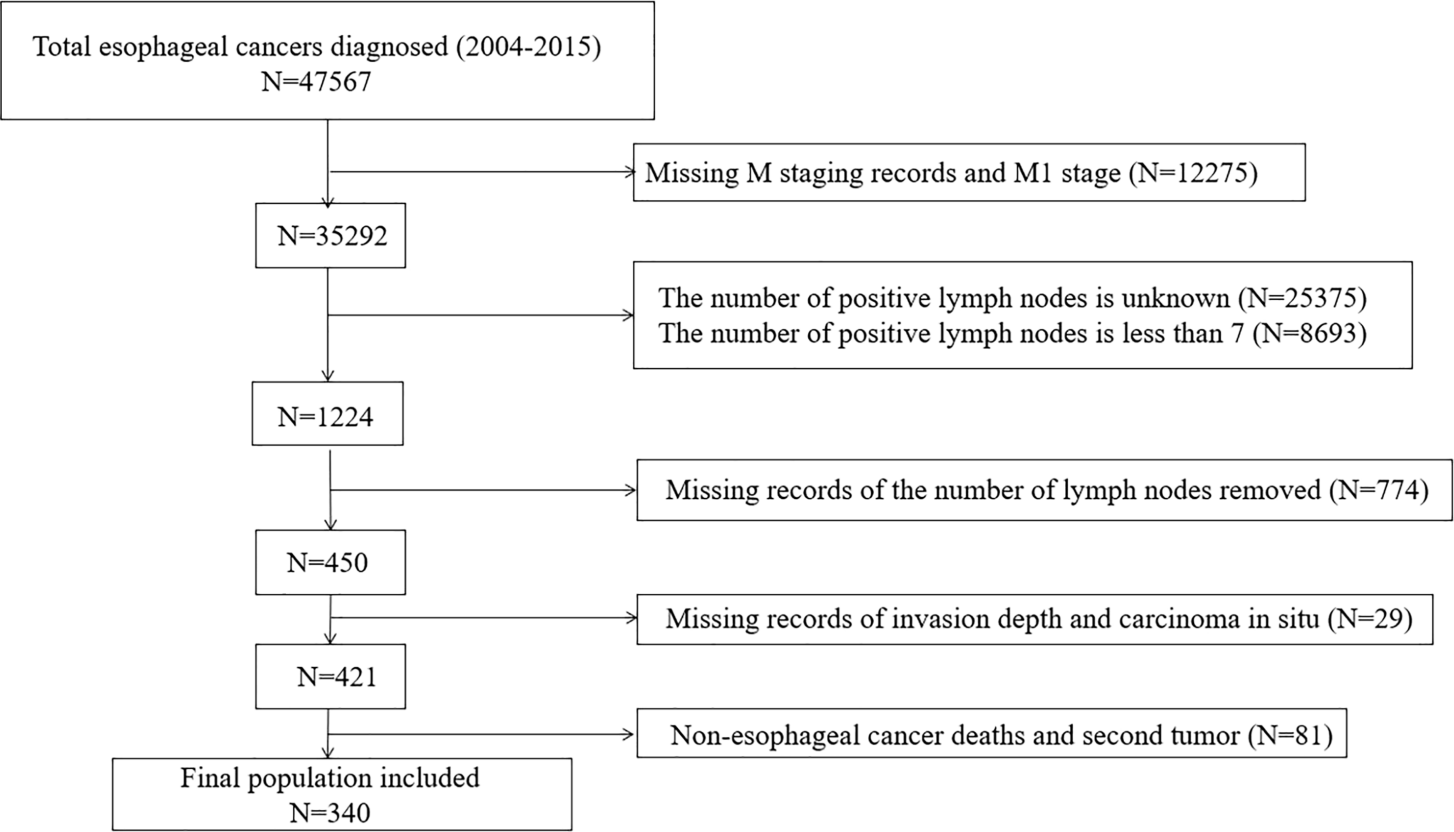
Study protocol design and application of inclusion/exclusion criteria in training cohort.

Data for this study were obtained from surveillance,epidemiology, and final results provided by SEER*Stat software (http://seer.cancer.gov/) as a training cohort. A total of 47,567patients diagnosed with EC between 2004 and 2015 were included to ensure at least 5 years of follow-up. The inclusion criteria were as follows: (1) complete clinical pathological information; (2) completesurvival times; (3) no distant metastasis; (4) retrieved at least 7 LNs; (5) at least 7 metastatic LNs; (6) death caused by EC; (7) no second tumor; (8) no carcinoma *in situ* (**Figure 1**). The depth of tumor invasion and the pN staging classification were rechecked according to the eighth edition of the AJCC staging system.

### Validation cohort

Patients diagnosed with EC from 2016-2017 were included in the SEER validation cohort. Patients for esophageal cancer who underwent radical surgery at the Department of Thoracic Surgery, Esophagus and Mediastinum of Harbin Medical University Cancer Hospital from June 2012 to July 2016 and were diagnosed as pN3 by postoperative pathology were used as the external validation cohort. Exclusion criteria used were similar to those used in SEER patients.

The clinicopathological data of the patients are stored in the case system of the Cancer Hospital Affiliated to Harbin Medical University, including sex, age, tumor size, tumor location, pT stage, pN stage, mLNs, RLNs, etc. The above contents are in compliance with the eighth edition of AJCC regulations. All patients were followed up by telephone, E-mail or examination in the outpatient complex building of the Cancer Hospital Affiliated to Harbin Medical University after discharge.

### Statistical methods

Disease-specific survival (DSS) was defined as the time from curative surgery to the date of death caused by EC and presented as the mean ± standard deviation and 5-year DSS rate. Both external validation and SEER validation cohorts were evaluated using DSS. The relationship between mLNs and the number of retrieved lymph nodes (RLNs) and hazard ratios (HRs) by a restricted cubic spline model. The optimal cutoff value for DSS transfer LNs was determined using X-tile software (X-Tile version 3.6.1 Yale University, New Haven, CT). X-tile is a visual bioinformatics software based on Kaplan-Meier survival analysis and log-rank test to determine the optimal tangent point of biomarkers, group the dataset according to the best tangent point, and perform statistical analysis of survival differences between the two groups. In this study, we input data on the number of metastatic lymph nodes into the X-tile software and finalize the cut-off value for the number of metastatic lymph nodes and group them (15). Furthermore, The receiver operating characteristic curve (ROC) was used to evaluate the optimal cut-off values of mLNs/RLNs, and the optimal cutoff value of each mLNs/was analyzed by the “Youden index”, which was calculated by the sensitivity-(1-specificity). The maximum value of the index was the optimal cutoff value. Linear regression and scatterplots are plotted by GraphPad Prism8, using pearson correlation coefficients and a two-tailed test to assess the correlation between the number of mLNs and RLNs. The Kaplan–Meier method and the log-rank test assessed the effect of cutoff values on the mLNs on prognosis. The chi-square test and Fisher’s exact test were applied to analyze the relationship between clinicopathological characteristics of patients with pN3 subclassification. A Forest plot was used to show the effect of RLNs on prognosis. A Cox proportional hazards model calculated HRs and 95% confidence intervals (95% CI). In all analyses, *P*<0.05 was considered statistically significant. All analyses were performed statistically with R software (version 4.1.2) and SPSS (version 25 for Windows).

## Results

### Patient characteristics

Ultimately, a total of 340 EC patients were included in the training cohort, including 304 (89.4%) males and 36 (10.6%) females. The mean age was 62.57 (range 23-89). For pT stage, there were 10 (2.9%) pT1, 4 (7.1%) pT2, 270 (79.4%) pT3, and 36 (10.6%) pT4 (**Table 1**).

**Table 1:** Clinicopathological characteristics of patients.

Characteristics	Training cohort (N=340)	SEER validation cohort (N=33)	External validation cohort (N=33)	*P* value
Sex				0.059
Male	304 (89.4)	28 (84.8)	33 (100.0)	
Female	36 (10.6)	5 (15.2)	0 (0.0)	
Age				0.043
≤60	140 (41.2)	9 (27.3)	19 (57.6)	
>60	200 (58.8)	24 (72.7)	14 (42.4)	
Race				0.312
Black	15 (4.4)	0 (0.0)	–	
White	315 (92.6)	31 (93.9)		
Other	10 (2.9)	2 (6.1)		
Tumor location				**<0.001**
Lower third of esophagus	287 (84.4)	28 (84.8)	17 (51.5)	
Nonlower third of esophagus	53 (15.6)	5 (15.2)	16 (48.4)	
Histological type				**<0.001**
Adenocarcinoma	281 (82.6)	26 (78.8)	3 (9.1)	
Squamous cell carcinoma	25 (7.4)	4 (12.1)	30 (90.9)	
Others	34 (10.0)	3 (9.1)	0 (0.0)	
Grade				<0.001
G1	26 (7.6)	5 (15.2)	11 (33.3)	
G2	91 (26.8)	9 (27.3)	14 (42.4)	
G3	223 (65.6)	19 (57.6)	8 (24.2)	
Tumor size(cm)				0.428
≤4	148 (43.5)	11 (33.3)	16 (48.5)	
>4	192 (56.5)	22 (66.7)	17 (51.5)	
pT stage				0.309
pT1	10 (2.9)	2 (6.1)	2 (6.1)	
pT2	24 (7.1)	2 (6.1)	5 (15.2)	
pT3	270 (79.4)	26 (78.8)	25 (75.8)	
pT4	36 (10.6)	3 (9.1)	1 (3.0)	
mLNs, median	9	10	9	0.348
Radiation therapy				**<0.001**
None	139 (40.9)	12 (36.4)	29 (87.9)	
Preoperative	118 (34.7)	15 (45.5)	1 (3.0)	
Postoperative	83 (24.4)	6 (18.2)	3 (9.1)	

Statistically significant P values are in bold (P<0.05).

A total of 33 EC patients were included in the SEER validation cohort, including 28 (84.8%) males and 5 (15.2%) females. The mean age was 65.36 (range 30-85). For pT stage, there were 2 (6.1%) pT1, 2 (6.1%) pT2, 26 (78.8%) pT3, and 3 (9.1%) pT4 (**Table 1**).

A total of 33 EC patients were included in the external validation cohort, including 100 (100.0%) males and 0 (0.0%) females. The mean age was 59.85 (range 45-80). For pT stage, there were 2 (6.1%) pT1, 5 (15.2%) pT2, 25 (75.8%) pT3, 1 (3.0%) pT4 (**Table 1**).

### Designing the subclassification of pN3 stage

**Figure 2:**
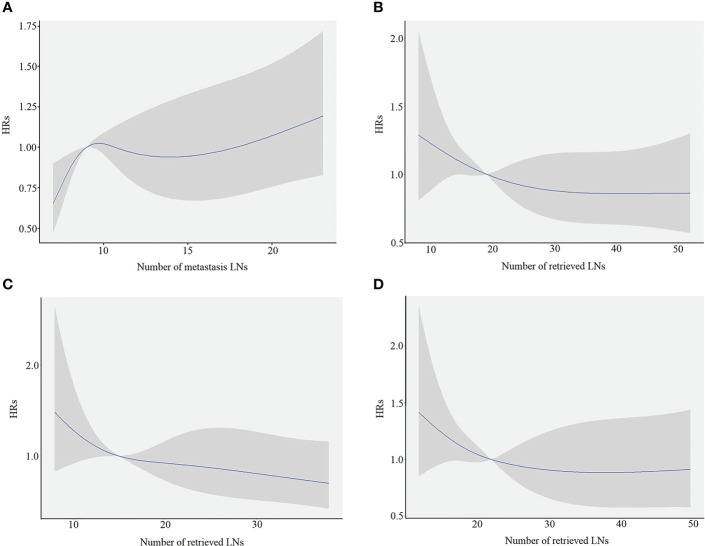
The restricted cubic spine model in training cohort. The blue line represents the estimated hazard ratios (HRs), and the shaded area is the 95% confidence interval (CI). **(A)** The association between the number of metastasis LNs and HRs. **(B)** The association between the number of retrieved LNs and the HRs for overall patients. **(C)** The association between the number of retrieved LNs and the HRs for pN3-I. **(D)** The association between the number of retrieved LNs and the HRs for pN3-II.

A restricted cubic spline analysis was performed to evaluate the association between the number of mLNs and HRs. The smooth curve showed that HRs increased with the increase in mLNs (**Figure 2A**). Furthermore, considering that mLNs are potentially affected by RLNs, the association between RLNs and HRs was further explored. The smooth curve showed that HRs decreased with the increase in RLNs (**Figure 2B**). The trends for pN3-I and pN3-II were also evaluated. The smooth curve showed that for pN3-I, HRs gradually decrease with the increase in RLNs. However, for the pN3-II, the curve reached a plateau when RLNs exceeded 30, although HRs continued to decrease with the increase in RLNs (**Figures 2C, D****)**.

**Figure 3:**
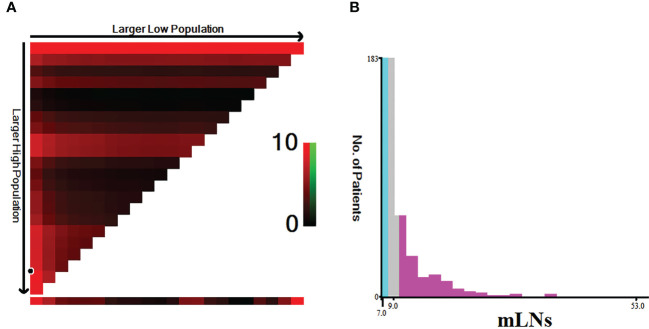
Estimation of the cutoff value of mLNs using X-tile software in training cohort. **(A)** X-tile plots based on the number of MLNs. **(B)** The optimal cut-off point is stressed by the gray, cyan and pink panels. The X-axis represents all potential cut-points from low to high (left to right) that define a low subset, whereas the Y-axis represents cut-points from high to low (top to bottom), that define a high subset. The arrows represent the direction in which the low subset (X-axis) and the high subset (Y-axis) increase in size. Red coloration of cut-points indicates an inverse correlation with survival, whereas greencoloration represents direct associations.

Because a nonlinear relationship between mLNs and HRs was found in patients with pN3 stage disease, X-tile software was used to determine the differences in survival among these patients. The X-tile software showed that the cutoff value of mLNs was 9 (**Figure 3**). Subsequently, the occurrence of 7-9 mLNs was defined as pN3-I, and the occurrence of more than 9 mLNs was defined as pN3-II. There were 183 (53.8%) pN3-I and 157 (46.2%) pN3-II (**Table 2**). The results showed that the two subclassifications were only statistically associated with the number RLNs (*P*<0.001) and the number of RLNs is higher in pN3-II patients than in pN3-I patients (**Table 2**).

**Table 2:** Comparison of clinicopathological characteristics between pN3-I and pN3-Ⅱ subclassifications in training cohort.

Characteristics	pN3-I (n=183)	pN3-II (157)	*P* value
Sex			0.894
Male	164 (89.6)	140 (89.2)	
Female	19 (10.4)	17 (10.8)	
Age			0.603
<=60	73 (39.9)	67 (42.7)	
>60	110 (60.1)	90 (57.3)	
Race			0.382
Black	10 (5.5)	5 (3.2)	
White	166 (90.7)	149 (94.9)	
Other	7 (3.8)	3 (1.9)	
Tumor location			0.449
Lower third of esophagus	157 (85.8)	130 (82.8)	
Nonlower third of esophagus	26 (14.2)	27 (17.2)	
Histological type			0.169
Adenocarcinoma	145 (79.2)	136 (86.6)	
Squamous cell carcinoma	15 (8.2)	10 (6.4)	
Others	23 (12.6)	11 (7.0)	
Grade			
G1	14 (7.7)	12 (7.6)	
G2	58 (31.7)	33 (21.0)	
G3	111 (60.7)	112 (71.3)	
Tumor size			0.885
≤4	79 (43.2)	69 (43.9)	
>4	104 (56.8)	88 (56.1)	
pT stage			0.333
pT1	7 (3.8)	3 (1.9)	
pT2	14 (7.7)	10 (6.4)	
pT3	146 (79.8)	124 (79.0)	
pT4	14 (7.7)	20 (12.7)	
RLNs, median	15	22	**<0.001**
Radiation therapy			0.416
None	69 (37.7)	70 (44.6)	
Preoperative	68 (37.2)	50 (31.8)	
Postoperative	46 (25.1)	37 (23.6)	

Statistically significant P values are in bold (P<0.05).

### Survival analysis of pN3 subclassification

**Figure 4:**
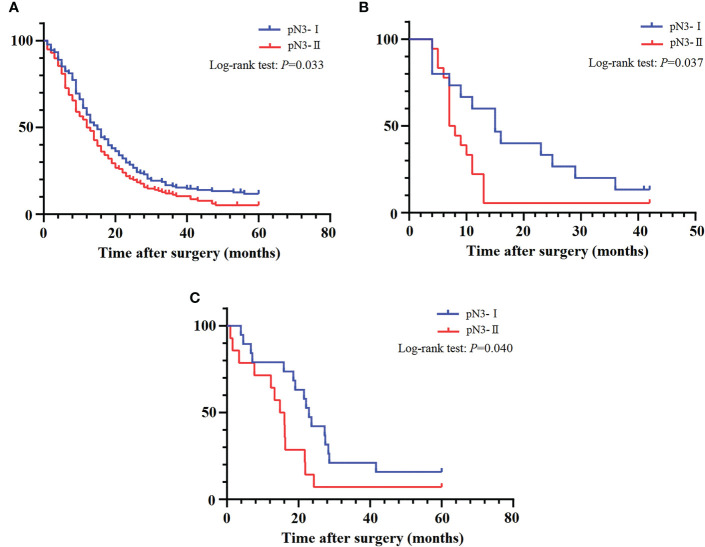
The DSS curves of pN3 patients. **(A)** Training cohort. **(B)** SEER validation cohort. **(C)** External validation cohort.

The survival curve showed that the 5-year DSS rates of pN3-I and pN3-II were 11.7% and 5.2% (20.66 ± 1.33, 16.86 ± 1.21, *P*=0.033), respectively (**Figure 4A**).

**Figure 5:**
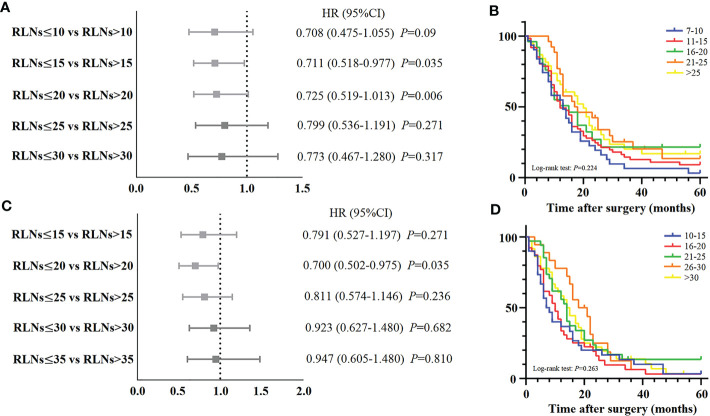
**(A, C)** Forest plot for the HRs of different subgroups of examined LNs stratified by pN3-I and pN3-II patients in training cohort. **(B)** The DSS curves of pN3-I patients according to the number of retrieved LNs in training cohort. **(D)** The DSS curves of pN3-II patients according to the number of retrieved LNs in training cohort.

Meanwhile, we also explored the benefit of RLNs on the 5-year DSS rate of patients. For pN3-I and pN3-II, the results showed no significant impact of increased RLNs on patient survival (**Figures 5A, C**). For pN3-I, 5-year DSS rates for patients with 7-10, 11-15, 16-20, 21-25, or >25 RLNs were 3.2%, 9.1%, 21.6%, 13.0%, 16.4%, respectively (16.26 ± 2.48, 18.95 ± 2.15, 22.46 ± 4.39, 24.47 ± 3.46, 23.22 ± 3.15, *P*=0.224) (**Figure 5B**). For pN3-II, 5-year DSS rates for patients with 10-15, 16-20, 21-25, 26-30, >30 RLNs were 3.3%, 3.2%, 13.5%, 6.3%, 3.5% (14.50 ± 2.77, 13.72 ± 2.14, 19.59 ± 3.10, 19.45 ± 2.31, 17.60 ± 2.31, *P*=0.263) (**Figure 5D**). This suggests that increasing RLNs may not prolong the prognosis of patients.

**Figure 6:**
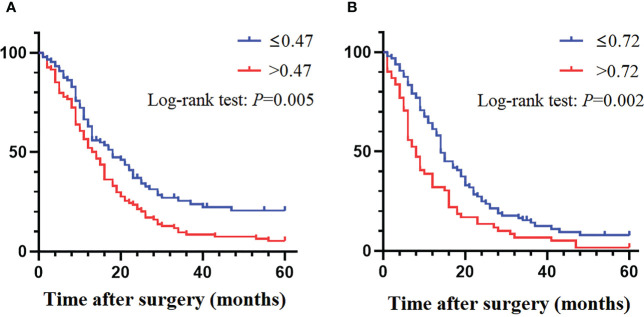
The DSS curves of pN3 subclassification based on mLNs/RLNs. **(A)** pN3-I. **(B)** pN3-II.

Therefore, in order to predict the prognosis of pN3 subclassification, we used ROC to calculate the cut-off values of mLNs/RLNs. The ROC showed that the cut-off values of mLNs/RLNs for pN3-I and pN3-II were 0.47 and 0.72.For pN3-I, the five-year DSS rate of mLNs/RLNs ≤0.47 and >0.47 were 20.1% and 5.3% (24.59 ± 2.22, 17.32 ± 1.51, *P*=0.005) (**Figure 6A**). For pN3-II, the five-year DSS rate of mLNs/RLNs ≤ 0.72 and >0.72 were 7.8% and 1.7% (19.64 ± 1.65, 12.56 ± 1.61, *P*=0.002) (**Figure 6B**).

### Staged migration

**Figure 7:**
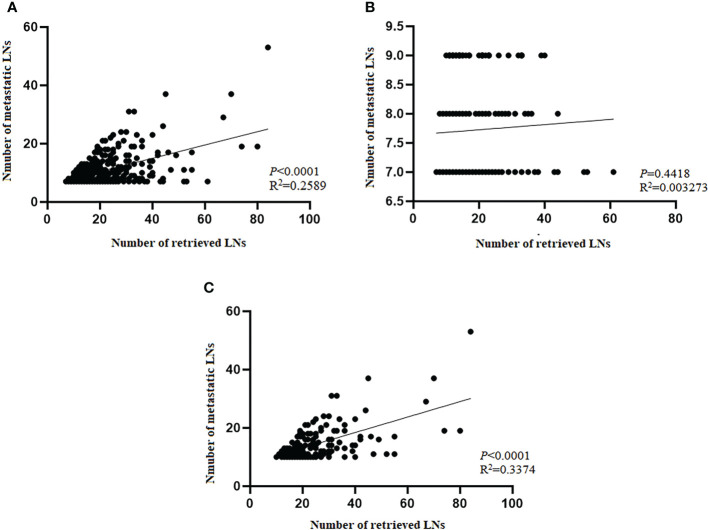
Scatter plot and linear regression analysis of the number of metastatic lymph nodes and positive lymph nodes in training cohort. **(A)** Total patients. **(B)** pN3-I. **(C)** pN3-II. Pearson’s correlation coefficient and two-tailed test to assess the correlation between the number of mLNs and RLNs.

To explore the effect of RLNs on mLNs, further investigation into the potential association between RLNs and mLNs was performed. For all patients, the linear relationship indicated that mLNs increased with increasing RLNs (*P*<0.0001, R^2^ = 0.2589) (**Figure 7A**), indicating that more mLNs could be found with more RLNs. However, this trend was not reflected in pN3-I (*P*=0.4418, R^2^ = 0.003273). For pN3-II, mLNs increased with increasing RLNs (*P*<0.0001, R^2^ = 0.3374) (**Figures 7B, C****)**.

### Univariate and multivariate analysis of the prognosis of patients

Univariate and multivariate analyses of Cox hazards regression models were performed to identify independent risk factors associated with patient outcomes. Univariate analysis showed that age (*P*<0.001), histological type (*P*=0.028), and subclassification of pN3 (*P*=0.039) were statistically significant. Multivariate analysis showed that age (*P*<0.001), histological type (*P*=0.007), and subclassification of pN3 (*P*=0.006) were independent risk factors associated with patient prognosis (**Table 3**).

**Table 3:** Univariate and multivariate analyses of prognostic factors for pN3 esophageal cancer patients in training cohort.

Characteristics	Univariate analysis		Multivariate analysis	
	HR (95% CI)	*P* value	HR (95% CI)	*P* value
Sex		0.325		
Male	1			
Female	1.203 (0.833-1.736)			
Age	1.034 (1.022-1.046)	**<0.001**	1.035 (1.023-1.047)	**<0.001**
Race		0.949		
Black	1			
White	0.915 (0.524-1.596)	0.753		
Other	0.894 (0.382-2.090)	0.796		
Tumor location		0.673		
Lower third of esophagus	1			
Nonlower third of esophagus	1.070 (0.781-1.466)			
Histological type		**0.028**		**0.007**
Adenocarcinoma	1		1	
Squamous cell carcinoma	1.086 (0.708-1.666)	0.706	0.958 (0.623-1.474)	0.845
Others	1.663 (1.146-2.415)	**0.007**	1.826 (1.250-2.669)	**0.002**
Grade		0.082		
G1	1			
G2	0.925 (0.566-1.514)	0.758		
G3	1.237 (0.781-1.959)	0.365		
Tumor size		0.763		
≤4	1			
>4	1.036 (0.823-1.304)			
pT stage		0.980		
pT1	1			
pT2	1.032 (0.454-2.343)	0.940		
pT3	1.045 (0.516-2.114)	0.903		
pT4	1.126 (0.517-2.451)	0.765		
RLNs	0.991 (0.981-1.001)	0.090		
Subclassification of pN3		**0.039**		**0.006**
pN3-I	1		1	
pN3-II	1.272 (1.012-1.598)		1.385 (1.098-1.746)	
Radiation therapy		0.103		
None	1			
Preoperative	0.808 (0.620-1.052)	0.113		
Postoperative	0.748 (0.559-1.000)	0.050		

Statistically significant P values are in bold (P<0.05).

In addition, considering the difference in survival between pN3-I and pN3-II, we analyzed the independent prognostic factors of pN3-I and pN3-II respectively. Of note, mLNs/RLNs are independent risk factors associated with prognosis in patients with pN3-I and pN3-II (*P*=0.001, *P*=0.014) (**Tables 4**, **5**).

**Table 4:** Univariate and multivariate analyses of prognostic factors for pN3-I esophageal cancer patients in training cohort.

Characteristics	Univariate analysis		Multivariate analysis	
	HR (95% CI)	*P* value	HR (95% CI)	*P* value
Sex		0.238		
Male	1			
Female	1.364 (0.824-2.259)			
Age	1.038 (1.022-1.055)	**<0.001**	1.011 (0.962-1.062)	0.669
Race		0.635		
Black	1			
White	1.165 (0.571-2.378)	0.674		
Other	1.651 (0.572-4.763)	0.354		
Tumor location		0.486		
Lower third of esophagus	1			
Nonlower third of esophagus	0.854 (0.548-1.331)			
Histological type		0.072		
Adenocarcinoma	1			
Squamous cell carcinoma	1.212 (0.696-2.109)	0.497		
Others	1.708 (1.073-2.717)	0.024		
Grade		0.374		
G1	1			
G2	0.653 (0.346-1.234)	0.189		
G3	0.772 (0.423-1.410)	0.400		
Tumor size		0.915		
≤4	1			
>4	1.017 (0.740-1.398)			
pT stage		0.994		
pT1	1			
pT2	1.111 (0.386-3.200)	0.845		
pT3	1.073 (0.439-2.626)	0.877		
pT4	1.137 (0.405-3.192)	0.808		
mLNs/RLNs	3.382 (1.591-7.188)	**0.002**	3.486(1.620-7.501)	**0.001**
Radiation therapy		0.119		
None	1			
Preoperative	0.772 (0.534-1.115)	0.167		
Postoperative	0.660 (0.439-0.993)	**0.046**		

Statistically significant P values are in bold (P<0.05).

**Table 5:** Univariate and multivariate analyses of prognostic factors for pN3-Ⅱ esophageal cancer patients in training cohort.

Characteristics	Univariate analysis		Multivariate analysis	
	HR (95% CI)	*P* value	HR (95% CI)	*P* value
Sex		0.912		
Male	1			
Female	1.031 (0.603-1.762)			
Age	1.030 (1.013-1.048)	**0.001**	1.027 (1.009-1.045)	**0.003**
Race		**0.043**		**0.027**
Black	1		1	
White	0.342 (0.137-0.850)	**0.021**	0.305 (0.121-0.767)	**0.012**
Other	0.196 (0.046-0.843)	**0.029**	0.186 (0.043-0.815)	**0.026**
Tumor location		0.090		
Lower third of esophagus	1			
Nonlower third of esophagus	1.474 (0.941-2.307)			
Histological type		0.118		
Adenocarcinoma	1			
Squamous cell carcinoma	0.965 (0.490-1.901)	0.919		
Others	1.978 (1.032-3.793)	**0.040**		
Grade		**0.034**		0.171
G1	1		1	
G2	1.357 (0.622-2.963)	0.443	1.206 (0.544-2.673)	0.645
G3	2.043 (0.994-4.198)	0.052	1.654 (0.801-3.418)	1.174
Tumor size		0.701		
≤4	1			
>4	1.068 (0.765-1.490)			
pT stage		0.976		
pT1	1			
pT2	0.768 (0.206-2.857)	0.693		
pT3	0.859 (0.272-2.710)	0.796		
pT4	0.895 (0.263-3.048)	0.859		
mLNs/RLNs	3.310 (1.482-7.397)	**0.004**	2.774 (1.228-6.268)	**0.014**
Radiation therapy		0.755		
None	1			
Preoperative	0.874 (0.594-1.285)	0.492		
Postoperative	0.892 (0.588-1.355)	0.593		

Statistically significant P values are in bold (P<0.05).

### Validation of pN3 subclassification

To verify the applicability of the pN3 subclassification, we performed validation in the patients of our institution with stage pN3 EC. The survival curve showed that the 5-year DSS rates of pN3-I and pN3-II in the SEER validation cohort were 13.3%, 5.6% (18.80 ± 3.35, 10.28 ± 1.93, *P*=0.037) (**Figure 4B**). The survival curve showed that the 5-year DSS rates of pN3-I and pN3-II in the external validation cohort were 15.8%, 7.1% (26.28 ± 3.96, 16.46 ± 3.75, *P*=0.040) (**Figure 4C**). This result shows that subclassification of pN3 is well validated in the validation cohort.

## Discussion

In this study, we designed a subclassification of pN3 staging for EC patients based on mLNs. The results showed that pN3-II had a worse prognosis than pN3-I, and that subclassification of pN3 was an independent risk factor related to patient outcomes. This also means that reasonable prognostic stratification will be further supplemented with traditional pN staging based on mLNs.

LNs status is considered one of the clinical variables impacting tumor dissemination after surgery because mLNs may reflect the malignant biological behaviors of EC cells, such as migration, lymphangiogenesis, and invasion (16, 17). Therefore, LNs status has been recognized as one of the most significant prognostic and recurrence predictors over the past decades. Recently, it has been found that the number of mLNs can provide comprehensive information about the metastasis of LNs, which is important in predicting prognosis.

Studies have shown that patients with 7 or more mLNs suffer from a poor prognosis with a fairly low 5-year survival rate of only 9.5% (5). The seventh edition of the AJCC staging system classified EC patients with 7 mLNs as pN3 stage. In addition, the eighth edition of staging retains the classification of N staging compared to the seventh edition of the staging. Obviously, in the context of the eighth edition of staging, classification based on the number of lymph node metastases remains important for patient prognosis. Meanwhile, this subtype also had a prognostic impact on different pT substages. Even in the early stage of the disease, the final pTNM stage of patients with pN3 stage was classified into Stage IV. Obviously, patients in Stage pT1N3M0 still belong to the high-risk group. In view of the low proportion and the extremely severe tumor burden in these patients, pN3 patients may have a similarly poor survival rate without considering the number of metastases in mLNs. Because previous studies have mostly focused on the overall survival of EC patients, this study only included patients who underwent curative surgery for EC-induced death to evaluate the survival effect of mLNs on pN3 patients as accurately as possible. Our analysis of the SEER database found that mLNs ranged from 7-53, which means that patients with different degrees of disease progression may be included in this large range. To evaluate the prognostic impact of mLNs on patients with pN3 stage, a smooth curve between HRs and mLNs was drawn. As a result, a nonlinear trend showed that risks increase rapidly with increasing mLNs and eventually reach a plateau. The results showed that the risk of death in patients with pN3 stage disease is not constant or increases with the number of mLNs, suggesting that there may be potential differences in survival among these patients.

In addition, in the context of the eighth edition of staging, Xi et al. classified the pN2 stage in more detail according to the number of metastatic lymph nodes, and could accurately predict the prognosis of patients (18). This further suggests that for traditional pTNM stage, there may still be significant differences in prognosis in patients, even at the same stage. Therefore, granularizing pN staging based on the number of metastatic lymph nodes can help to further differentiate the risk stratification of patients, help clinicians better understand the heterogeneous process of disease, and more comprehensively assess the biological behavior of tumors. This also provides a good theoretical basis for our research (18).

Based on the above considerations, we used X-tile software to explore differences in survival among patients with pN3 stage and determine the optimal cutoff value. Although only 340 patients who died from EC were included in the analysis, significant differences in survival were observed when the two subgroups were distinguished with the optimal cutoff value applied. The results showed that the 5-year DSS rate of patients with 7-9 mLNs (pN3-I) was significantly higher than that of patients with ≥10 mLNs (pN3-II) (11.7% vs. 5.2%). These results also indicated that there are potential high-risk pN3 stage patients. Notably, we included patients who received preoperative radiotherapy, which differs from previous studies (19, 20), because adjuvant radiotherapy has become a standard treatment modality for patients with advanced EC (21). Metastatic LNs can still be found in EC resection specimens treated with radiotherapy, although radiotherapy induces LN interstitial fibrosis and depletion, resulting in LN shrinkage (22). Groth et al. also included EC patients after adjuvant radiotherapy and found that EC patients with neoadjuvant radiotherapy had fewer LNs than those who did not complete neoadjuvant radiotherapy (23). Obviously, the status of LNs is critical for patient outcomes with or without adjuvant radiotherapy. Our study showed that the novel pN3 classification is an independent risk factor associated with patient prognosis, while the SEER database also includes multiple ethnicities of EC patients, such as white, black, Indian, Pacific Islander, etc., which is worthy of promotion and use in clinical practice.

Related studies on RLNs have found that the long-term survival of patients can be prolonged with more RLNs (24, 25). However, no consensus has been reached on the cutoff values of the number of RLNs in different studies. The National Cancer Data Base (NCDB) found that at least 20 to 25 LNs be removed intraoperatively, whereas the international multicenter study suggests that surgeons remove at least 23 LNs (26, 27). Although the optimal cutoff value of RLNs may not be uniform, the potential survival benefit of increasing RLNs found the same trend in different studies. Even for early EC, when the number of RLNs exceeds 14, the 5-year cancer-specific survival rate of patients increased by 11% compared with < 14 RLNs (28). If the LNs are insufficiently dissected, it may lead to poor outcomes and the need for adjuvant therapy. How increasing RLNs can prolong the long-term survival of patients can be explained as follows. First, extensive LN metastases of EC patients with local or distant tumor burden will increase. In contrast, extensive LN resection can eliminate potential metastases through local control of LNs and modulate immunity to improve the survival rate of patients with LN metastases (29, 30). Moreover, increasing RLNs helps to find more positive LNs or potential micrometastatic LNs (3), which means that stage migration may occur when RLNs are insufficient (31). Stage migration may not ensure accurate pN staging, so subsequent treatment may be biased in assessing prognosis.

The present study found that there was no correlation between RLNs and mLNs for patients with the pN3-I subtype, while the results of the survival curve showed that increasing RLNs could prolong the long-term survival of patients without statistical significance. This may be due to the small number of patients, but this trend indicates that prolonging the 5-year DSS rate in patients with the pN3-I subtype by increasing the number of RLNs may be influenced by the actual therapeutic benefit of RLNs rather than by the phenomenon of stage migration. Furthermore, for pN3-II patients, the linear relationship showed a significant correlation between mLNs and RLNs, which indicates that there was stage migration in pN3-II patients. Although the survival curve did not have statistical significance, the trend showed that when RLNs reached 21 to 25, the 5-year DSS rate of patients was prolonged. Additional RLNs do not improve patient prognosis, which may be, partly due to the inability to distinguish between stage migration and the influence of treatment benefit with LN resection. This conclusion also needs to be verified by expanding the sample size. Another reason may be related to LN dissection. Some scholars believe that more aggressive tumors may cause a stronger immune response, leading to regional lymph node hyperplasia, thereby enhancing the detectability of lymph nodes (24, 32). We found that pN3-II has a higher number of RLNs than pN3-I, which is also in line with the view of previous studies. Therefore, the reason for the higher number of pN3-II RLNs may be related to a stronger immune response, which may lead to regional lymph node hyperplasia and is more conducive to lymph node detection. Pathological examination of surgical and excised specimens is helpful in finding more LNs (24), while extensive LN dissection may affect the patient’s immune function (33). Therefore, we speculate that the reason why additional RLNs do not significantly improve the prognosis of pN3-II may be related to the effect of extensive LNs resection on patient immunity. This speculation also needs to be verified later. Overall, these results further suggest that pN3-I and pN3-II might belong to the two distinct subtypes of disease progression.

He et al. used mLNs/RLNs to predict the prognosis of EC patients and found that their predictive performance was better than traditional pN staging, and it was helpful to select individualized postoperative treatment options (34). At the same time, in the study of He et al., mLNs/RLNs are independent risk factors related to patient prognosis, which further indicates that mLNs/RLNs are of great significance for the prognosis of EC patients, indicating that mLNs/RLNs help to avoid staged migration and achieve accurate prognosis prediction. Therefore, based on the survival difference between pN3-I and pN3-II. To further accurately assess patient prognosis, we used ROC to calculate the cut-off values of mLNs/RLNs for pN3-I and pN3-II, and found that mLNs/RLNs can accurately predict the prognosis of pN3-I and pN3-II, and mLNs/RLNs are independent risk factors related to patient prognosis. This fully suggests that the use of mLNs/RLNs will help to further accurately predict the prognosis of patients after distinguishing high-risk subgroups of pN3.

In conclusion, the findings of the present study showed that there are still some high-risk patients with poor prognosis even in the same stage of pN3 EC patients with heavier tumor burden. It is recommended that the pN3 stage be more specific and substaged according to the number of mLNs reported by postoperative pathology to improve the prognosis of these patients, which can individualize the risk stratification of patients, accurately identify high-risk patients and adopt a comprehensive treatment plan.

### Research limitations

As a retrospective, single-center study, the present study has several limitations. Firstly, the incidence of pN3 is rare, the validation cohorts samples are small, and although our results have been validated. The results still need to be verified by multi-center, large samples. Furthermore, the SEER database lacks clinicopathological information of some patients, such as lymphatic tumor thrombus, occult lymph node metastasis, and tumor marker levels. These factors may affect the mLNs of patients, which makes it difficult to assess the impact of different clinicopathological features on RLNs. Meanwhile, our endpoint was DSS, some factors of death unrelated to disease will remain unknown. Last, this study included patients in the SEER database spanning 10 years, during which adjuvant therapy modalities changed, such as different chemotherapy regimens and the application of immunotherapy. Thus, it may not be possible to assess the sensitivity of the pN3 stage subclass to chemotherapy and immunotherapy.

## Conclusion

We proposed a novel pN3 subclassification based on mLNs. And the pN3 subclassification can well distinguish the difference in survival of EC patients. Furthermore, the subclassification of pN3 was well validated.

## Data availability statement

The original contributions presented in the study are included in the article/supplementary material. Further inquiries can be directed to the corresponding author.

## Ethics statement

All programs followed were according to the ethical standards of the Human Subjects Responsibility Committee (institutions and countries), as well as the 1964 Helsinki Declaration and subsequent editions. This research was approved by the Ethics Committee of the Harbin Medical University Cancer Hospital (Registration number 2021-49-IIT).

## Author contributions

KM and HW designed and conceived this project, they contributed equally to this work. KM and HW interpretated and analysised the data. JM revised the manuscript for important intellectual content, KM, HW, CF, and XJ participated in the patient information collention. All authors contributed to the article and approved the submitted version.

## References

[1] Sung H Ferlay J Siegel RL Laversanne M Soerjomataram I Jemal A. Global cancer statistics 2020: GLOBOCAN estimates of incidence and mortality worldwide for 36 cancers in 185 countries. CA Cancer J Clin (2021) 71(3):209–49. doi: 10.3322/caac.21660 33538338

[2] Rice TW Ishwaran H Hofstetter WL Schipper PH Kesler KA Law S. Esophageal cancer: associations with (pN+) lymph node metastases. Ann Surg (2017) 265(1):122–9. doi: 10.1097/SLA.0000000000001594 PMC5405457 28009736

[3] Rizk NP Ishwaran H Rice TW Chen LQ Schipper PH Kesler KA. Optimum lymphadenectomy for esophageal cancer. Ann Surg (2010) 251(1):46–50. doi: 10.1097/SLA.0b013e3181b2f6ee 20032718

[4] Semenkovich TR Yan Y Subramanian M Meyers BF Kozower BD Nava R. A clinical nomogram for predicting node-positive disease in esophageal cancer. Ann Surg (2021) 273(6):e214–21. doi: 10.1097/SLA.0000000000003450 PMC6940556 31274650

[5] Shao Y Geng Y Gu W Ning Z Huang J Pei H. Assessment of lymph node ratio to replace the pN categories system of classification of the TNM system in esophageal squamous cell carcinoma. J Thorac Oncol (2016) 11(10):1774–84. doi: 10.1016/j.jtho.2016.06.019 27393473

[6] Hwang JY Chen HS Hsu PK Chao YK Wang BY Huang CS. A propensity-matched analysis comparing survival after esophagectomy followed by adjuvant chemoradiation to surgery alone for esophageal squamous cell carcinoma. Ann Surg (2016) 264(1):100–6. doi: 10.1097/SLA.0000000000001410 26649580

[7] Hsu PK Wu YC Chou TY Huang CS Hsu WH. Comparison of the 6th and 7th editions of the American joint committee on cancer tumor-node-metastasis staging system in patients with resected esophageal carcinoma. Ann Thorac Surg (2010) 89(4):1024–31. doi: 10.1016/j.athoracsur.2010.01.017 20338302

[8] Talsma K van Hagen P Grotenhuis BA Steyerberg EW Tilanus HW van Lanschot JJ. Comparison of the 6th and 7th editions of the UICC-AJCC TNM classification for esophageal cancer. Ann Surg Oncol (2012) 19(7):2142–8. doi: 10.1245/s10434-012-2218-5 PMC3381120 22395974

[9] Kim HI Cheong JH Song KJ An JY Hyung WJ Noh SH. Staging of adenocarcinoma of the esophagogastric junction: comparison of AJCC 6th and 7th gastric and 7th esophageal staging systems. Ann Surg Oncol (2013) 20(8):2713–20. doi: 10.1245/s10434-013-2898-5 23456315

[10] Zhang J Li H Zhou L Yu L Che F Heng X. Modified nodal stage of esophageal cancer based on the evaluation of the hazard rate of the negative and positive lymph node. BMC Cancer (2020) 20(1):1200. doi: 10.1186/s12885-020-07664-w 33287741 PMC7720494

[11] Rice TW Ishwaran H Ferguson MK Blackstone EH Goldstraw P. Cancer of the esophagus and esophagogastric junction: an eighth edition staging primer. J Thorac Oncol (2017) 12(1):36–42. doi: 10.1016/j.jtho.2016.10.016 27810391 PMC5591443

[12] Tan Z Ma G Yang H Zhang L Rong T Lin P. Can lymph node ratio replace pn categories in the tumor-node-metastasis classification system for esophageal cancer? J Thorac Oncol (2014) 9(8):1214–21. doi: 10.1097/JTO.0000000000000216 25157776

[13] Jang HJ Lee HS Kim MS Lee JM Zo JI. Patterns of lymph node metastasis and survival for upper esophageal squamous cell carcinoma. Ann Thorac Surg (2011) 92(3):1091–7. doi: 10.1016/j.athoracsur.2011.03.093 21704967

[14] Lagergren J Mattsson F Zylstra J Chang F Gossage J Mason R. Extent of lymphadenectomy and prognosis after esophageal cancer surgery. JAMA Surg (2016) 151(1):32–9. doi: 10.1001/jamasurg.2015.2611 26331431

[15] Camp RL Dolled-Filhart M Rimm DL. X-Tile: a new bio-informatics tool for biomarker assessment and outcome-based cut-point optimization. Clin Cancer Res (2004) 10(21):7252–9. doi: 10.1158/1078-0432.CCR-04-0713 15534099

[16] Mori D Yamasaki F Shibaki M Tokunaga O. Lateral peritumoral lymphatic vessel invasion can predict lymph node metastasis in esophageal squamous cell carcinoma. Mod Pathol (2007) 20(6):694–700. doi: 10.1038/modpathol.3800786 17464319

[17] Wang Y Zhu L Xia W Wang F. Anatomy of lymphatic drainage of the esophagus and lymph node metastasis of thoracic esophageal cancer. Cancer Manag Res (2018) 10:6295–303. doi: 10.2147/CMAR.S182436 PMC6267772 30568491

[18] Xi K Yu H. Proposed modification of the pN2 classification of the 8th edition AJCC staging system for esophageal squamous cell carcinoma: a preliminary study based on the Chinese population. J Oncol (2021) 2021:8871884. doi: 10.1155/2021/8871884 33777143 PMC7972858

[19] Altorki NK Zhou XK Stiles B Port JL Paul S Lee PC. Total number of resected lymph nodes predicts survival in esophageal cancer. Ann Surg (2008) 248(2):221–6. doi: 10.1097/SLA.0b013e31817bbe59 18650631

[20] Bollschweiler E Baldus SE Schröder W Schneider PM Hölscher AH. Staging of esophageal carcinoma: length of tumor and number of involved regional lymph nodes. are these independent prognostic factors? J Surg Oncol (2006) 94(5):355–63. doi: 10.1002/jso.20569 16967455

[21] Ajani JA D'Amico TA Bentrem DJ Chao J Corvera C Das P. Esophageal and esophagogastric junction cancers, version 2.2019, NCCN clinical practice guidelines in oncology. J Natl Compr Canc Netw (2019) 17(7):855–83. doi: 10.6004/jnccn.2019.0033 31319389

[22] Habr-Gama A Perez RO Proscurshim I Rawet V Pereira DD Sousa AH. Absence of lymph nodes in the resected specimen after radical surgery for distal rectal cancer and neoadjuvant chemoradiation therapy: what does it mean? Dis Colon Rectum (2008) 51(3):277–83. doi: 10.1007/s10350-007-9148-5 18183463

[23] Groth SS Virnig BA Whitson BA DeFor TE Li ZZ Tuttle TM. Determination of the minimum number of lymph nodes to examine to maximize survival in patients with esophageal carcinoma: data from the surveillance epidemiology and end results database. J Thorac Cardiovasc Surg (2010) 139(3):612–20. doi: 10.1016/j.jtcvs.2009.07.017 19709685

[24] van der Werf LR Dikken JL van Berge Henegouwen MI Lemmens V Nieuwenhuijzen GAP Wijnhoven BPL. A population-based study on lymph node retrieval in patients with esophageal cancer: results from the Dutch upper gastrointestinal cancer audit. Ann Surg Oncol (2018) 25(5):1211–20. doi: 10.1245/s10434-018-6396-7 PMC5891559 29524046

[25] Wu LL Zhong JD Zhu JL Kang L Huang YY Lin P. Postoperative survival effect of the number of examined lymph nodes on esophageal squamous cell carcinoma with pathological stage T1-3N0M0. BMC Cancer (2022) 22(1):118. doi: 10.1186/s12885-022-09207-x 35090428 PMC8800278

[26] Samson P Puri V Broderick S Patterson GA Meyers B Crabtree T. Extent of lymphadenectomy is associated with improved overall survival after esophagectomy with or without induction therapy. Ann Thorac Surg (2017) 103(2):406–15. doi: 10.1016/j.athoracsur.2016.08.010 PMC5362278 28024648

[27] Peyre CG Hagen JA DeMeester SR Altorki NK Ancona E Griffin SM. The number of lymph nodes removed predicts survival in esophageal cancer: an international study on the impact of extent of surgical resection. Ann Surg (2008) 248(4):549–56. doi: 10.1097/SLA.0b013e318188c474 18936567

[28] Wang Y Zhang X Zhang X Liu-Helmersson J Zhang L Xiao W. Prognostic value of the extent of lymphadenectomy for esophageal cancer-specific survival among T1 patients. BMC Cancer (2021) 21(1):403. doi: 10.1186/s12885-021-08080-4 33853577 PMC8045314

[29] Jia Y Wang H Wang Y Wang T Wang M Ma M. Low expression of Bin1, along with high expression of IDO in tumor tissue and draining lymph nodes, are predictors of poor prognosis for esophageal squamous cell cancer patients. Int J Cancer (2015) 137(5):1095–106. doi: 10.1002/ijc.29481 25683635

[30] Gnerlich J Jeffe DB Deshpande AD Beers C Zander C Margenthaler JA. Surgical removal of the primary tumor increases overall survival in patients with metastatic breast cancer: analysis of the 1988-2003 SEER data. Ann Surg Oncol (2007) 14(8):2187–94. doi: 10.1245/s10434-007-9438-0 17522944

[31] Feinstein AR Sosin DM Wells CK. The will Rogers phenomenon. stage migration and new diagnostic techniques as a source of misleading statistics for survival in cancer. N Engl J Med (1985) 312(25):1604–8. doi: 10.1056/NEJM198506203122504 4000199

[32] Märkl B. Stage migration vs immunology: the lymph node count story in colon cancer. World J Gastroenterol (2015) 21(43):12218–33. doi: 10.3748/wjg.v21.i43.12218 PMC4649108 26604632

[33] Zhou J Zhang WW Wu SG He ZY Sun JY Wang Y. The impact of examined lymph node count on survival in squamous cell carcinoma and adenocarcinoma of the uterine cervix. Cancer Manag Res (2017) 9:315–22. doi: 10.2147/CMAR.S141335 PMC5522663 28761376

[34] He Z Wu S Li Q Lin Q Xu J. Use of the metastatic lymph node ratio to evaluate the prognosis of esophageal cancer patients with node metastasis following radical esophagectomy. PloS One (2013) 8(9):e73446. doi: 10.1371/journal.pone.0073446 24039944 PMC3767826

